# The Problematic Behaviour Scale (PBS-5): A brief measure for the population-level screening of non-substance-bound addictive behaviours and Swiss national prevalence rates

**DOI:** 10.1556/2006.2025.00120

**Published:** 2026-03-20

**Authors:** Sebastian Mader, Simon Marmet

**Affiliations:** 1 Swiss Federal Office of Public Health FOPH, Bern, Switzerland; 2 University of Bern, Bern, Switzerland

**Keywords:** Assessment of Criteria for Specific Internet-use Disorders, ACSID-11, Problematic Behaviour Scale, PBS-5, addictive disorders, screening instruments, psychometric characteristics, receiver operator characteristics regression, prevalence rates, addictive behaviors, gaming, social media use, gambling, pornography use, compulsive buying-shoping behaviour

## Abstract

**Background and aims:**

The Assessment of Criteria for Specific Internet-use Disorders (ACSID-11; Müller et al. 2022) offers a promising instrument to screen addictive disorders in population-wide surveys based on ICD-11 criteria. We evaluated this scale in the Swiss context and propose the shorter Problematic Behaviour Scale (PBS-5).

**Methods:**

We included ACSID-11 in the Swiss survey “Health and Lifestyle” (SHL) 2023 (*n* = 5,995) in German, French and Italian for social media use, shopping, gaming, gambling, and pornography use. We assessed psychometric characteristics of PBS-5. Classification quality and cut-off values of PBS-5 for prevalences of problematic behaviour were determined by multiple Receiver Operator Characteristic regressions of the Patient Health Questionnaire-4 (PHQ-4) on PBS-5. Including PBS-5 and PHQ-4 in the SHL 2025 (*n* = 5,818) allowed to investigate trends and the intertemporal stability of the cut-off values.

**Results:**

PBS-5 showed a one-dimensional factorial structure, and excellent internal consistency. External validity and classification quality was limited. Prevalence rates of problematic behaviours as a percentage of regular users in Switzerland in 2025 were: social media use: 27.7%, shopping: 13.6%, gaming: 21.0%, gambling: 2.5%, pornography use: 22.2%. Since 2023, these prevalences have increased except for gambling. Problematic social media use peaked among young women and gaming among young men. Problematic gaming, and pornography use were more frequent among men. Problematic shopping was observed more frequently among subjects with low education.

**Discussion and conclusions:**

PBS-5 is a promising brief screening tool for addictive behaviours. Further validation is recommended.

## Introduction

Problematic usage of the internet (PUI) refers to excessive or poorly controlled engagement with online technologies, characterized by impaired control and shifts in priorities away from important responsibilities. Such patterns of use may result in adverse familial, social, educational, and occupational consequences (Zare-Bidoky et al., 2025). The European Network for Problematic Usage of the Internet (Fineberg et al., 2022) has proposed eight major forms of problematic internet-use behavior subsumed under PUI: online gaming disorder, online gambling disorder, online buying-shopping disorder, online compulsive sexual behavior disorder, problematic usage of social media, cyberchondria, cyberbullying, and digital hoarding. Currently, gaming disorder (6C51) and gambling disorder (6C50) are included explicitly in the ICD-11 in the category ‘disorders due to addictive behaviours’ (World Health Organization, 2025). The remaining disorders due to addictive behaviours could potentially be subsumed into the ICD-11 category ‘other specified disorders due to addictive behaviours’ (6C5Y) or ‘disorders due to addictive behaviours, unspecified’ with potentially similar diagnostic requirements (e.g., Brand et al., 2020, 2025; Oelker, Rumpf, Brand, & Müller, 2024) – be it online or offline. In DSM-5-TR, only gambling disorder is currently included as a behavioral addiction, internet gaming disorder is additionally included for research purposes, but not as a clinical diagnosis (American Psychiatric Association, 2013).

The Assessment of Criteria for Specific Internet-use Disorders (ACSID-11) introduced and initially tested by Müller et al. (2022) in a German study offers a valuable 11-item instrument to screen several different non-substance-bound addictive behaviours in population-wide surveys with the same set of items. Based on the diagnostic criteria of the International Classification of Diseases 11th Revision (ICD-11), ACSID-11 covers the three main dimensions of disorders due to addictive behaviours ‘impaired control’, ‘increased priority’, and ‘continuation/escalation’ that lead to marked distress and functional impairment in life. Additionally, it includes the latter dimensions of ‘functional impairment in daily life’ and ‘marked distress’. Given the heterogeneity of screening instruments in the field, ACSID-11 reacts to the need to develop a harmonized instrument based on the same diagnostic criteria and utilizing the same items bridging different disorders due to addictive behaviours. This provides a common aetiological framework of addictive behaviours (for further discussion see e.g., Griffiths, 2005) and guarantees comparability (Müller et al., 2022; Oelker et al., 2024).

In 2021, Müller et al. (2022) administered ACSID-11 to a convenience sample of almost 1,000 active internet users aged 16–69 years and recruited via an access panel service provider from the German-speaking area. Müller et al. (2022) assessed the frequency and intensity of problems associated with the following five behaviours: social media use, shopping, gaming, gambling, and pornography use. Applying confirmatory factor analysis, Müller et al. (2022) found that the assumed four-factorial structure (impaired control, increased priority, continuation/escalation, and functional impairment) showed a better model fit as compared to a unidimensional solution. ACSID-11 showed very high internal consistency (Cronbach's *α* = .90 for gaming to *α* = .95 for online gambling). Correlations of ACSID-11 with a very brief instrument to screen symptoms of depression and anxiety (the Patient Health Questionnaire-4 – PHQ-4; Kroenke, Spitzer, Williams, & Löwe, 2009) were moderate (*r* = .26 for online pornography use to *r* = .35 for social-networks use). Müller et al. (2022) concluded that ACSID-11 “may be a useful and economic instrument for studying various behavioral addictions with the same items and improving comparability” (Müller et al., 2022: 427).

Oelker et al. (2024) further validated ACSID-11 using the same five behaviours as Müller et al. (2022) in a German convenience sample of more than 1,500 active internet users aged 18–69 years against established screening instruments of specific internet-use disorders and measures of mental health. Furthermore, Oelker et al. (2024) proposed a cut-off-value to determine prevalence rates of pathological behaviour. The cut-off was calculated as follows: a score of one was given to individuals who stated that they experienced the problem with the behaviour at least often in one out of the items of the respective theoretical dimension. Then the sum score over the four dimensions was calculated. If a maximum of four out of four was reached, the individuals were coded as showing pathological behaviour. Oelker et al. (2024) found high internal consistency of ACSID-11, comparable to Müller et al. (2022) and the other administered established screening instruments. Moreover, the authors found medium correlations of ACSID-11 with established scales (*r* = .46 for online buying-shopping to *r* = .61 for gaming), which alludes to the fact that the scales differ in the diagnostic criteria they cover. Furthermore, the authors showed that the dichotomized ACSID-11 sum score at the proposed cut-off was moderately correlated with measures of mental health (*r* = .23 to *r* = .43 for symptoms of depression and anxiety). Hence, the authors concluded that ACSID-11 is a reliable and valid instrument to screen non-substance-bound behavioural disorders.

Further studies tested ACSID-11 in Asian countries: Huang et al. (2025), and Yang et al. (2023) provide evidence that also a Traditional Chinese, and a Thai version of ACSID-11 showed high reliability and validity in Taiwan and Hong Kong, and Thailand respectively. Moreover, Huang et al. (2025) and Yang et al. (2023) showed in a confirmatory factor analysis that a one-factorial solution had good and similar fit index values as compared to the four-factorial solution.

However, to be applied to the Swiss context with German, French, and Italian as the main languages and to other countries, ACSID-11 needs to be further tested and investigated: ACSID-11 leaves room for abbreviation and even more efficient implementation in population-wide surveys. Therefore, we propose a short 5-item version of the scale we term Problematic Behaviour Scale (PBS-5) and compare its performance with the longer PBS-10 with 10 items.

## Methods

### Study 1: psychometric properties, and cut-offs based on 2023 data

#### Data

In Study 1, we included an adapted 10-item version of ACSID-11 (PBS-10) in the Swiss nationally representative cross-sectional survey study “Health and Lifestyle” 2023 (SFOPH, 2024; *n* = 5,995). The study consists of a random sample of the permanent resident population of Switzerland aged at least 15 years (mean age = 48.5 years). The survey was fielded in German, French, and Italian using computer-assisted web interviewing (CAWI).

PBS-10 was only administered to participants that reported the respective behaviour at least weekly in the last 30 days (see Table 1 for the pre-query on the frequency of behaviours). Like the previous studies, we investigated the following five behaviours: social media use, shopping, gaming, gambling, and pornography use. We generalized the scale for offline and online behaviour. In their pre-query, Müller et al. (2022) asked to tick the activities with at least occasional use in the past 12 months. Instead, we asked for the frequency of use in the last 30 days, used a more detailed scale, and abbreviated the explanations of the behaviours. Tables 2 and 3 show the items of the adapted ACSID-11. We asked for the frequency of the occurrence of symptoms associated with the behaviours on a five-point scale from ‘never’ to ‘always’. The original scale by Müller et al. (2022) used a four-point scale from ‘never’ to ‘often’. For brevity and because of its similarity with the item IP1, item IP2 was left out of the original scale. Since in the previous literature the performance of the scales for the frequency, and for the intensity of the occurrence of symptoms were comparable, and for the sake of reducing participants' burden, we focused on frequency and omitted repeating the scale for intensity.

**Table 1.** Items of the pre-query on the frequency of behaviours

Item		Question wording in			
		English (translation from German)	German	French	Italian
Introduction		The following are various activities.	Im Folgenden geht es um verschiedene Aktivitäten.	Ce qui suit concerne différentes activités.	Di seguito sono menzionate diverse attività.
		Please indicate how often you have used these activities in the last 30 days. It does not matter whether they took place online or offline.	Bitte geben Sie an, wie oft Sie diese Aktivitäten in den letzten 30 Tagen genutzt haben. Dabei spielt es keine Rolle, ob diese online oder offline stattfanden.	Veuillez indiquer à quelle fréquence vous avez eu recours aux activités suivantes les 30 derniers jours (en ligne ou hors ligne).	Indichi quante volte le ha svolte negli ultimi 30 giorni, non ha importanza se online oppure offline.
1		Use of social networks (e.g., Facebook, WhatsApp, Instagram, Tik-Tok, etc.)	Nutzung von sozialen Netzwerken (z.B. Facebook, WhatsApp, Instagram, Tik-Tok usw.)	Réseaux sociaux (p. ex. Facebook, WhatsApp, Instagram, Tik-Tok, etc.)	Utilizzo di social network (p. es. Facebook, WhatsApp, Instagram, Tik-Tok, ecc.)
2		Shopping (purchase of goods, price comparisons, product search)	Shopping (Kauf von Waren, Preisvergleiche, Produktsuche)	Shopping (achats, comparaison de prix, recherche de produits)	Shopping (acquisto di beni, confronto di prezzi, ricerca di prodotti)
3		Gaming (computer, video and online games)	Gaming (Computer-, Video- und Online-Spiele)	Jeux vidéo (sur ordinateur, sur console ou en ligne)	Gaming (videogiochi, giochi al computer e online)
4		Money games and games of chance (gambling, e.g., casino games, lotteries)	Geld- und Glücksspiele (Gambling, z.B. Casino-Spiele, Lotterien)	Jeux de hasard et d'argent (p. ex. jeux de casino, loteries)	Giochi in denaro e giochi d'azzardo (p. es. giochi da casinò, lotterie)
5		Use of pornography	Nutzung von Pornografie	Consultation de contenus pornographiques	Consumo di pornografia
Answer cate-gories	1	never	nie	jamais	mai
	2	less than once a week	seltener als einmal in der Woche	moins d'une fois par semaine	meno di una volta alla settimana
	3	once a week	einmal in der Woche	une fois par semaine	una volta alla settimana
	4	2–4 days a week	an 2–4 Tagen in der Woche	2 à 4 jours par semaine	2–4 giorni alla settimana
	5	5–6 days a week	an 5–6 Tagen in der Woche	5 à 6 jours par semaine	5–6 giorni alla settimana
	6	once a day	einmal pro Tag	une fois par jour	una volta al giorno
	7	several times a day	mehrmals pro Tag	plusieurs fois par jour	più volte al giorno

**Table 2.** Wording of the introduction of the Problematic Behaviour Scale (PBS-5)

	Wording in			
	English (translation from German)	German	French	Italian
General introduction	Please read the following questions carefully in relation to the activities you use. From the five possible answers, select the one that best describes how often you have had each experience in the last 30 days.	Bitte lesen Sie die folgenden Fragen im Zusammenhang mit den von Ihnen genutzten Aktivitäten aufmerksam durch. Wählen Sie aus den fünf Antwortmöglichkeiten diejenige aus, die am besten beschreibt, wie häufig Sie die jeweiligen Erfahrungen in den letzten 30 Tagen gemacht haben.	Veuillez lire avec attention les questions suivantes en rapport avec les activités que vous avez pratiquées. Parmi les cinq réponses possibles, choisissez la fréquence la plus adaptée pour décrire la situation vécue les 30 derniers jours.	Legga attentamente le seguenti domande in relazione alle attività da Lei svolte. Tra le cinque opzioni di risposta scelga quella che corrisponde maggiormente alla frequenza con cui ha vissuto la rispettiva situazione negli ultimi 30 giorni.
	There are no right or wrong answers.	Hierbei gibt es weder richtige noch falsche Antworten.	Il n'y a pas de réponse juste ou fausse.	Non esistono risposte giuste o sbagliate.
Introduction to social media use	Now think about the activity “Social media use” (e.g. Facebook, Instagram, Tik-tok, etc.).	Denken Sie nun an die Aktivität “Nutzung sozialer Medien” (z. B. Facebook, Instagram, Tik-tok usw.).	Pensez maintenant à l'activité “Utilisation des médias sociaux” (par exemple Facebook, Instagram, Tik-tok, etc.).	Ora pensi all'attività “Uso dei social media” (es. Facebook, Instagram, Tik-tok, ecc.).
Introduction to shopping	Now think about the activity “Shopping” (buying goods, comparing prices, searching for products).	Denken Sie nun an die Aktivität “Shopping” (Kauf von Waren, Preisvergleiche, Produktsuche).	Pensez maintenant à l'activité “Shopping” (achats, comparaison de prix, recherche de produits).	Ora pensi all'attività “Shopping” (acquisto di beni, confronto di prezzi, ricerca di prodotti).
Introduction to gaming	Now think about the activity “Gaming” (on the computer, on the console or online).	Denken Sie nun an die Aktivität “Gaming” (am Computer, auf der Konsole oder online).	Pensez maintenant à l'activité “Jeux vidéo” (sur ordinateur, sur console ou en ligne).	Ora pensi all'attività “Gaming” (videogiochi, giochi al computer o online).
Introduction to gambling	Now think about the activity “Gambling” (e.g. casino games, lotteries).	Denken Sie nun an die Aktivität “Geld- und Glücksspiel (Gambling)” (z.B. Casino-Spiele, Lotterien).	Pensez maintenant à l'activité “Jeux de hasard et d'argent” (p. ex. jeux de casino, loteries).	Ora pensi all'attività “Giochi di denaro e giochi d'azzardo” (p. es. giochi da casinò, lotterie).
Introduction to pornography use	Now think about the activity “Use of pornography”.	Denken Sie nun an die Aktivität “Nutzung von Pornografie”.	Pensez maintenant à l'activité “Consultation de contenus pornographiques”.	Ora pensi all'attività “Consumo di pornografia”.

**Table 3.** Items of the **Problematic Behaviour Scale (PBS-5)** as adapted from the Assessment of Criteria for Specific Internet-use Disorders (ACSID-11) scale (Müller et al., 2022)

Item		Question wording in			
		English (translation from German)	German	French	Italian
IC1^#^		How often have you had problems controlling the activity (e.g., start, duration, intensity, situation, end)?	Wie häufig haben Sie Probleme damit gehabt, die Aktivität zu kontrollieren (z. B. Beginn, Dauer, Intensität, Situation, Ende)?	Avez-vous eu des problèmes à contrôler cette activité (p. ex. début, durée, intensité, situation, arrêt)?	Con quale frequenza ha avuto difficoltà a tenere traccia dell'attività (ad es. inizio, durata, intensità, situazione, fine)?
IC2		How often have you felt the desire to stop or restrict the activity because you noticed you were using it too much?	Wie häufig haben Sie den Wunsch verspürt, mit der Aktivität aufzuhören oder sie einzuschränken, weil Sie bemerkt haben, dass Sie diese zu viel nutzen?	Avez-vous ressenti le besoin d'arrêter l'activité ou de la limiter car vous avez remarqué que vous la pratiquiez trop?	Con quale frequenza ha avvertito il desiderio di interrompere l'attività o di limitarla perché si è reso/a conto di praticarla in maniera eccessiva?
IC3*		**How often have you tried to stop or restrict the activity and failed with it?**	**Wie häufig haben Sie versucht, mit der Aktivität aufzuhören oder sie einzuschränken, und sind damit gescheitert?**	**Avez-vous cherché à arrêter l'activité ou à la limiter, sans succès?**	**Con quale frequenza ha cercato di interrompere l'attività o di limitarla, fallendo però nel tentativo?**
IP1		How often have you given the activity an increasingly higher priority than other activities or interests in your daily life?	Wie häufig haben Sie der Aktivität einen zunehmend höheren Stellenwert gegeben als anderen Tätigkeiten oder Interessen in Ihrem Alltag?	Avez-vous accordé une importance de plus en plus grande à cette activité au quotidien par rapport à d'autres activités ou centres d'intérêt?	Con quale frequenza l'importanza da Lei attribuita all'attività è aumentata sempre più rispetto a quella attribuita ad altre attività o ad altri interessi della Sua vita quotidiana?
IP2		Have you lost interest in other activities you used to enjoy because of the activity?	Haben Sie aufgrund der Aktivität das Interesse an anderen Tätigkeiten verloren, die Sie früher gerne gemacht haben?		
IP3*		**How often have you neglected or given up other activities or interests that you used to enjoy because of the activity?**	**Wie häufig haben Sie aufgrund der Aktivität andere Tätigkeiten oder Interessen vernachlässigt oder aufgegeben, die Sie früher gerne gemacht haben?**	**Avez-vous négligé ou abandonné d'autres activités ou centres d'intérêt que vous pratiquiez avec plaisir avant en raison de cette activité?**	**Con quale frequenza, a causa dell'attività, ha trascurato o abbandonato altri interessi o altre attività a cui si dedicava volentieri in passato?**
CE1		How often have you continued or increased the activity even though it has threatened or caused you to lose a relationship with someone important to you?	Wie häufig haben Sie die Aktivität fortgesetzt oder gesteigert, obwohl Sie dadurch eine Beziehung zu einem Ihnen wichtigen Menschen gefährdet oder verloren haben?	Avez-vous poursuivi ou augmenté cette activité bien qu'elle ait mis en danger ou mis fin à une relation avec une personne qui vous était importante?	Con quale frequenza ha proseguito o intensificato l'attività sebbene ciò abbia messo a repentaglio o interrotto il rapporto con una persona per Lei importante?
CE2		How often have you continued or increased the activity even though it caused you problems at school/in training/at work?	Wie häufig haben Sie die Aktivität fortgesetzt oder gesteigert, obwohl Sie dadurch Probleme in der Schule/in der Ausbildung/im Beruf bekommen haben?	Avez-vous poursuivi ou augmenté cette activité bien qu'elle vous ait causé des problèmes à l'école/dans votre formation/au travail ?	Con quale frequenza ha proseguito o intensificato l'attività sebbene ciò Le abbia causato problemi a scuola/nella formazione/al lavoro?
CE3*		**How often have you continued or increased the activity even though it caused you physical or mental complaints/diseases?**	**Wie häufig haben Sie die Aktivität fortgesetzt oder gesteigert, obwohl Sie dadurch körperliche oder psychische Beschwerden/Erkrankungen bekommen haben?**	**Avez-vous poursuivi ou augmenté cette activité bien qu'elle vous ait causé des troubles/maladies physiques ou psychiques ?**	**Con quale frequenza ha proseguito o intensificato l'attività sebbene ciò Le abbia causato malattie/disturbi fisici o psichici?**
FI1*		**If you think about all areas of your life, how often has your life been noticeably affected by the activity?**	**Wenn Sie an alle Ihre Lebensbereiche denken, wie häufig war Ihr Leben durch die Aktivität spürbar beeinträchtigt?**	**Lorsque vous pensez à tous les aspects de votre vie, celle-ci a-t-elle été fortement impactée par cette activité ?**	**Pensando a tutti gli ambiti della Sua vita, con quale frequenza l'attività ha interferito tangibilmente con essa?**
MD1*		**If you think about all areas of your life, how often did the activity cause you suffering?**	**Wenn Sie an alle Ihre Lebensbereiche denken, wie häufig verursachte Ihnen die Aktivität Leid?**	**Lorsque vous pensez à tous les aspects de votre vie, cette activité vous a-t-elle fait souffrir ?**	**Pensando a tutti gli ambiti della Sua vita, con quale frequenza l'attività Le ha procurato sofferenze?**
Answer cate-gories	1	never	niemals	jamais	mai
	2	rarely	selten	rarement	raramente
	3	sometimes	manchmal	quelquefois	talvolta
	4	often	oft	souvent	spesso
	5	always	immer	toujours	sempre

*Note:* IC = impaired control, IP = increased priority, CE = continuation/escalation, FI = functional impairment, MD = marked distress. All items are part of the original ACSID-11 scale. Only the 5 items marked with an * and in bold font are used for PBS-5. Items marked with a ^#^ have been modified as compared to Müller et al. (2022) for comprehensibility. The questions were only administered to participants that reported the respective behaviour at least weekly in the last 30 days.

For the selection of the 5 items for the Problematic Behaviour Scale (PBS-5; see Table 3 in bold font) the theoretically most difficult item per theoretical dimension was taken (IC3, IP3, and CE3) plus the single items for functional impairment (FI1) and marked distress (MD1). PBS-5 is the sum index of these 5 items. High reported frequencies for IC3, IP3, and CE3 indicate the presence of severe subjectively perceived problems with the control of the use of the behaviour and represent the essence of the meaning of impaired control, increased priority, and continuation/escalation, while the other items IC1, IC2, IP1, IP2, CE1, and CE2 indicate less problem severity.

Empirical evidence indicates that non-substance-bound addictive behaviours are associated with psychological distress and mental problems, and are inversely correlated with subjective well-being (e.g., Müller et al., 2022). Hence, to evaluate the external validity of PBS-5, we followed Müller et al. (2022) and analyzed its association with the Patient Health Questionnaire-4 (PHQ-4; Kroenke et al., 2009), a very brief instrument to screen core symptoms of depression and anxiety. PHQ-4 shows reliability and validity across diverse populations (e.g., Adzrago, Walker, & Williams, 2024; Kroenke et al., 2009; Wicke, Krakau, Löwe, Beutel, & Brähler, 2022). Its brevity makes inclusion of PHQ-4 in surveys practical.

#### Statistical analysis

In Study 1, we assessed the factorial structure of the PBS-5 using orthogonal varimax-rotated exploratory factor analysis (principal component analysis (PCA)) based on polychoric correlations for ordinal data. The criterion for factor extraction was an eigenvalue greater than 1. Internal consistency was analyzed using Cronbach's alpha. To determine cut-offs of the normalized score of PBS-5 for each behaviour, we conducted multiple Receiver Operator Characteristic (ROC) regressions of PHQ-4 on PBS-5. ROC Regression enables the determination of the cut-off of the normalized score of PBS-5 (0–100) with the best model fit (area under the curve – AUC) simultaneously minimizing Type I (*α*) and Type II (*β*) error of classification while controlling for potential confounders (here: gender, age, education, and survey language). Put differently, ROC regression simultaneously maximizes the specificity (1- α) and the sensitivity (1- β) of a classification task (Janes & Pepe, 2009; Pepe, 2000, 2003). These analyses were conducted using listwise deletion of missings. For a detailed description of the variables see Table A1 and for a correlation matrix of the frequencies of behavioural conduct and PBS-5 see Table A2 in the Appendix.

### Study 2: replication with 2025 data

#### Data

To test the intertemporal reliability of psychometric characteristics of PBS-5 and PHQ-4 as well as of the ROC regression-based cut-off determination approach, in study 2, we included the five items of the PBS-5, and the four items of the PHQ-4 in the Swiss nationally representative cross-sectional survey study “Health and Lifestyle” 2025 (SFOPH, 2025; *n* = 5,818) two years after Health and Lifestyle 2023. The setup, procedures and materials of Health and Lifestyle (2025) were similar to its 2023 edition. In addition, this allows trend analysis of prevalence rates.

#### Statistical analysis

The same methods were applied to the data of Health and Lifestyle (2025). For a detailed description of the variables see Table S1 in the Supplementary material.

#### Ethics

Informed consent of the participants of Study 1 and Study 2 was obtained within the postal invitation letter for the survey and on the first page of the online questionnaire. Since this study is based on a voluntary and anonymized survey, it does not fall under the Human Research Act according to local (Swiss) law. Hence, it does not require authorization from an ethics committee.

## Results

### Study 1

#### Exploratory factor analysis and internal consistency

The PCA retained a one-factorial solution for the three survey languages separately and for all cases together with high factor loadings (see Table 4, and Tables A3–7 in the Appendix). The share of the variance explained by this single factor ranges from 72% for social media to 93% for gambling. Internal consistency of the PBS-5 is sufficiently high, and varies from *α* = .83 for social media to *α* = .95 for gambling.

**Table 4.** The Problematic Behaviour Scale (PBS-5): exploratory factor analysis and internal consistency

Empirical factor	Theoretical dimension		Item	Social media	Shopping	Gaming	Gambling	Porn
I	Impaired control	(1)	How often have you tried to stop or restrict the activity and failed with it?	.81	.87	.85	.94	.85
	Increased priority	(2)	How often have you neglected or given up other activities or interests that you used to enjoy because of the activity?	.83	.92	.89	.96	.88
	Continuation/escalation	(3)	How often have you continued or increased the activity even though it caused you physical or mental complaints/diseases?	.85	.93	.89	.97	.90
	Functional impairment	(4)	If you think about all areas of your life, how often has your life been noticeably affected by the activity?	.88	.93	.91	.99	.92
	Marked distress	(5)	If you think about all areas of your life, how often did the activity cause you suffering?	.87	.94	.91	.96	.93
*n*				5,295	3,055	1,585	197	900
Percent of variance explained by empirical factor I				72.3	84.6	79.2	93.0	80.3
Cronbach's *α*				.83	.88	.87	.95	.84
PBS-5:			Mean	7.53	6.10	7.00	7.08	6.67
			SD	3.19	2.40	3.12	4.01	2.91
			Min	5	5	5	5	5
			Max	25	25	25	25	25

*Note:* Introduction: “Please read the following questions carefully in relation to the activities you use. From the five possible answers, select the one that best describes how often you have had each experience in the last 30 days. There are no right or wrong answers. Now think about the activity “Social media use”/“Shopping”/“Gaming”/“Gambling”/“Use of pornography”.” Answer categories: 1 = ”never”, 2 = ”rarely”, 3 = ”sometimes”, 4 = ”often”, 5 = ”always”. Numbers indicate factor loadings after orthogonal varimax-rotated exploratory factor analysis (principal component analysis (PCA)) based on polychoric correlations for ordinal data. The criterion for factor extraction is an eigenvalue greater than 1. For the detailed results by survey language (German, French, Italian) and behaviour see Tables A3–7 in the Appendix. An equivalent analysis based on Pearson's correlations yields substantially similar results.

PHQ-4 was used as external validation instrument of PBS-5. Table 5 shows the results of the PCA of PHQ-4 indicating a one-factorial solution with high factor loadings and percent of variance explained. Internal consistency of PHQ-4 is also high (*α* = .87). PHQ-4 is the sum index of the 4 items depicted in Table 5.

**Table 5.** The Patient Health Questionnaire-4 (PHQ-4; Kroenke et al., 2009: 615): Exploratory factor analysis and internal consistency

Empirical factor	Item	Over the last 2 week, how often have you been bothered by the following problems?	All	DE	FR	IT
I	(1)	Feeling nervous, anxious, or on edge	.90	.90	.91	.92
	(2)	Not being able to stop or control worrying	.88	.88	.90	.88
	(3)	Feeling down, depressed, or hopeless	.94	.94	.94	.95
	(4)	Little interest or pleasure in doing things	.88	.89	.88	.87
*N*			5,980	3,849	1,471	660
Percent of variance explained by empirical factor I			81.1	81.2	82.1	81.7
Cronbach's *α*			.87	.87	.87	.86
PHQ-4:		Mean	5.91	5.86	6.11	5.71
		SD	2.57	2.50	2.75	2.55
		Min	4	4	4	4
		Max	16	16	16	16

*Note:* Numbers indicate factor loadings after orthogonal varimax-rotated exploratory factor analysis (principal component analysis (PCA)) based on polychoric correlations for ordinal data. The criterion for factor extraction is an eigenvalue greater than 1. DE = German, FR = French, IT = Italian. The translations of the English scale can be found in SFOPH, (2024). Each item was measured on the following four-point scale: 1 = not at all, 2 = several days, 3 = more than half the days, 4 = nearly every day.

Since PCA is a data-reduction technique and does not explicitly model latent constructs, we carried out a Confirmatory Factor Analysis (CFA) for PBS-5 and PHQ-4 as a robustness check. The results of these Structural Equation Models (SEM) also support a one-factorial solution (see Figure S1, and Tables S2 and S3 of the SI for detailed results).

#### External validity

**Fig. 1.**
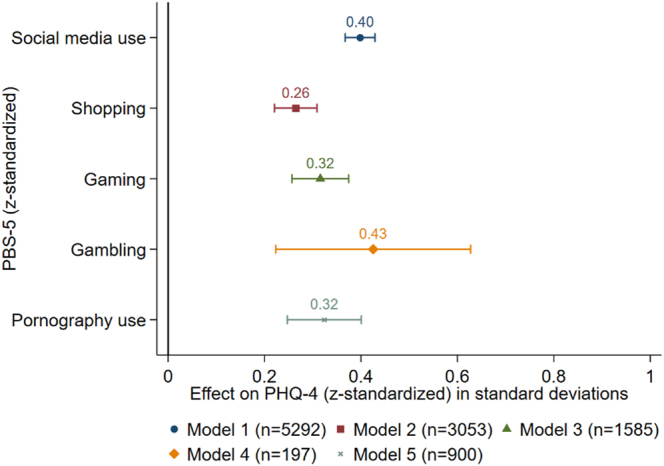
Multiple OLS regressions of PHQ-4 on PBS-5 *Note:* Coefficient plot of multiple OLS-regressions including 95% confidence intervals based on 100 bootstrap replications. Each model includes the PBS-5 of each activity (social media, shopping, gaming, gambling, or pornography) separately. Each model controls for gender, age, education and survey language. For the detailed results of the regressions see Table A8 in the Appendix.

The external validation analysis shows that for 1 standard deviation (SD) higher values of PBS-5 PHQ-4 is higher between .26 SD for shopping and .43 SD for gambling (all *p* <.001) (see Fig. 1). Each OLS regression controls for gender, age, education and survey language.

#### Cut-offs

**Fig. 2.**
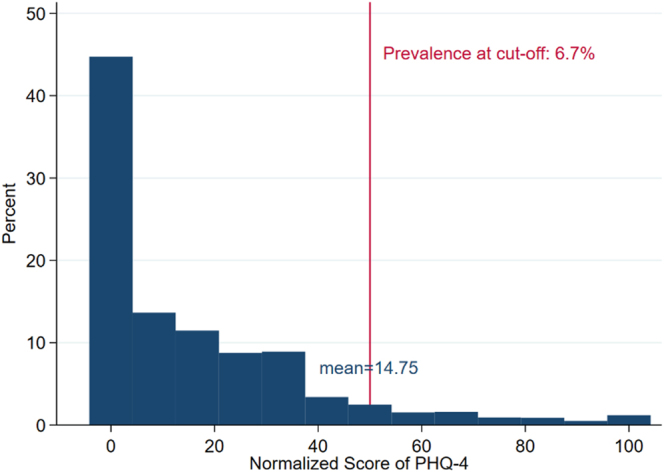
Distribution of PHQ-4 *Note: n* = 5,980.

To determine cut-offs of PBS-5 for each of the five behaviours, ROC-regression requires to dichotomize PHQ-4 as well. We decided to cut the normalized score of PHQ-4 ranging from 0 to 100 in two halves. Hence, participants with normalized values of PHQ-4 ≤50 receive the value of 0 (scant symptoms of depression and anxiety), and individuals with PHQ-4 >50 are assigned the value of 1 (ample symptoms of depression and anxiety). This procedure retains a prevalence of 6.7% of the Swiss resident population aged at least 15 years with ample symptoms of depression and anxiety (see Fig. 2).

Based on PHQ-9 (Kroenke, Spitzer, & Williams, 2001), the results of the Swiss Health Survey 2022 indicate that 9.8 and 6.3% of the population aged 15 years and older suffer from moderate to severe symptoms of a depression and a generalized anxiety disorder respectively (SFSO, 2024). Hence, the cut-off we used for PHQ-4 appears quite reasonable.

**Fig. 3.**
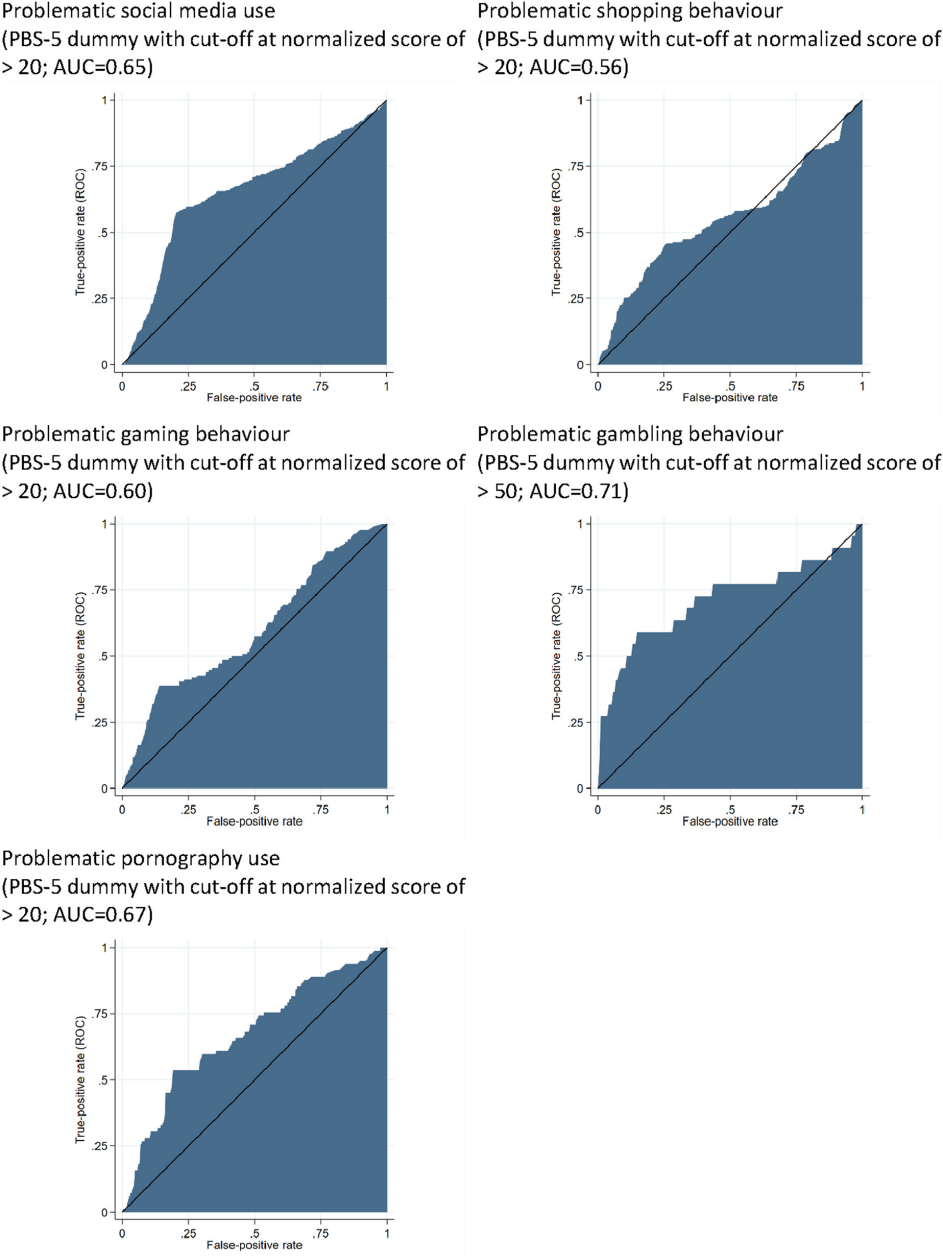
Area under the curve (AUC) of multiple ROC-regressions of PHQ-4 on PBS-5 (see Table 6)

ROC-regressions of this PHQ-4 dummy show that the highest AUCs for the tested cut-offs (20, 40, 50, 60, and 80) of the normalized PBS-5 scores (0–100) vary between AUC=.56 for shopping to AUC=.71 for gambling (see Table 6). The cut-offs are selected based on the highest behaviour-specific AUC. Hence, a cut-off of the normalized values of PBS-5 of 20 is proposed for social media use, shopping, gaming, and pornography use. A cut-off value of 50 is proposed for gambling. Figure 3 depicts the AUCs of the multiple ROC-regressions of these cut-offs.

**Table 6.** Multiple receiver operator characteristic (ROC) regressions of PHQ-4 on PBS-5

Model	(1)	(2)	(3)	(4)	(5)
Dependent variable	PHQ-4 (dummy with cut-off at >50)				
Independent variable	PBS-5 (dummy with cut-off at >20)	PBS-5 (dummy with cut-off at >40)	PBS-5 (dummy with cut-off at >50)	PBS-5 (dummy with cut-off at >60)	PBS-5 (dummy with cut-off at >80)
Social media	**0.65 (0.02)**	0.56 (0.02)	0.48 (0.02)	0.42 (0.02)	0.46 (0.10)
*n*	4,932	4,932	4,932	4,932	4,932
Shopping	**0.56 (0.03)**	0.50 (0.03)	0.47 (0.05)	0.43 (0.06)	
*n*	2,815	2,815	2,815	2,815	
Gaming	**0.60 (0.04)**	0.56 (0.05)	0.51 (0.05)	0.49 (0.06)	
*n*	1,451	1,451	1,451	1,451	
Gambling	0.62 (0.10)	0.61 (0.09)	**0.71 (0.11)**	0.66 (0.15)	0.63 (0.32)
*n*	175	175	175	175	175
Pornography	**0.67 (0.04)**	0.67 (0.05)	0.58 (0.07)	0.57 (0.08)	
*n*	818	818	818	818	

*Note:* Area under the curve (AUC) of multiple ROC-regressions including standard errors in parentheses based on 100 bootstrap replications. Each model includes the dummy of the normalized values of PBS-5 of each behaviour (social media, shopping, gaming, gambling, or pornography) separately. Each model controls for gender, age, education and survey language. AUCs in bold font indicate the selected cut-offs.

#### National prevalence rates of use frequency and problematic behaviour 2023

**Fig. 4.**
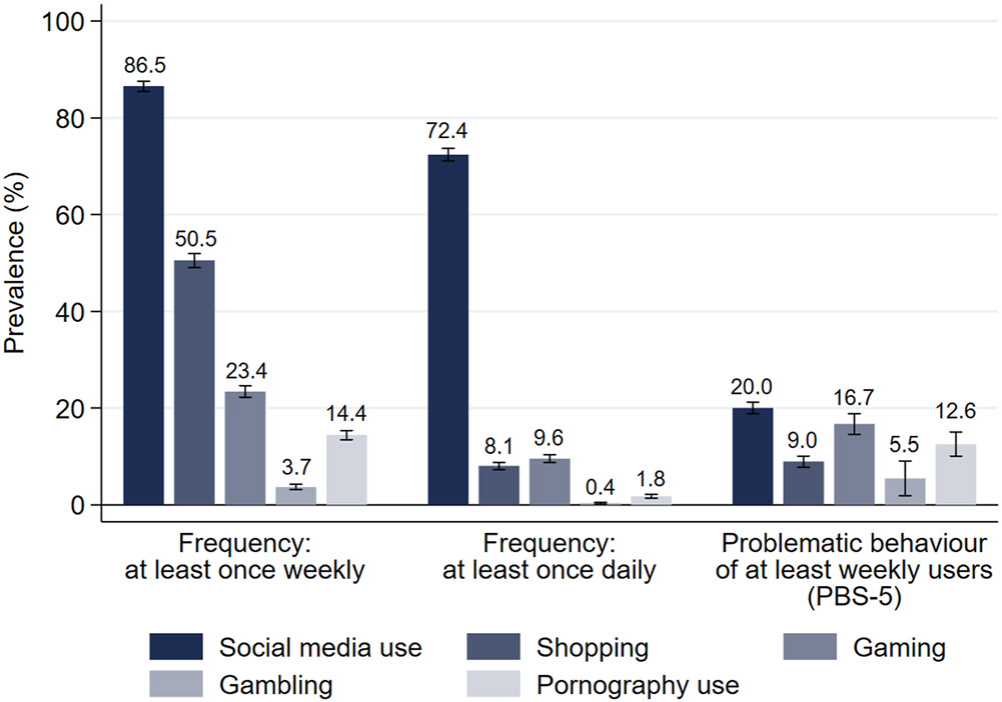
2023: Prevalences of the frequencies of behavioural conduct (within the last 30 days) and the Problematic Behaviour Scale (PBS-5; at least weekly users within the last 30 days) *Note:* Prevalences are nationally representative using sample weights. Whiskers indicate 95-% confidence intervals. For a more detailed description of the variables see Table A1 in the Appendix.

**Fig. A1.**
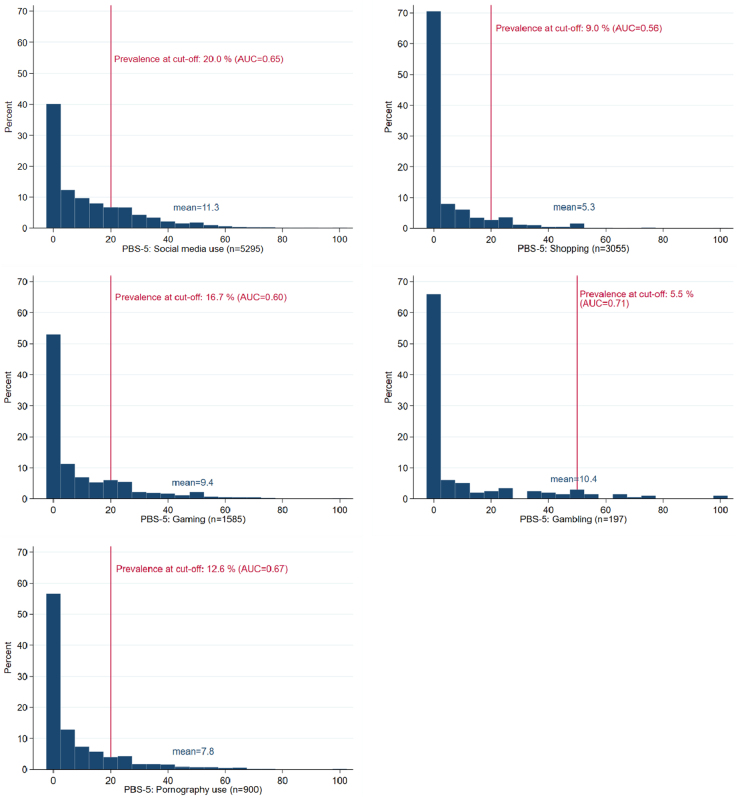
Health and Lifestyle 2023: Distributions of normalized scores (0–100) of PBS-5 by behaviour

Based on these cut-offs, prevalence rates of problematic behaviour as a percentage of regular users (at least weekly in the last 30 days) in Switzerland in 2023 were: social media use: 20.0%, gaming: 16.7%, pornography use: 12.6%, shopping: 9.0%, and gambling: 5.5% (see Fig. 4; for the distributions of normalized scores of PBS-5 by behaviour see Figure A1 in the Appendix). Figure 4 also depicts the frequencies of use by behaviour: Social media is used the most frequent, around 87% of the Swiss resident population aged at least 15 years report at least once weekly use in the last 30 days. 51% report at least weekly shopping, followed by 23% of at least weekly video gamers and 14% of pornography users. 4% report at least weekly gambling behaviour.

**Fig. 5.**
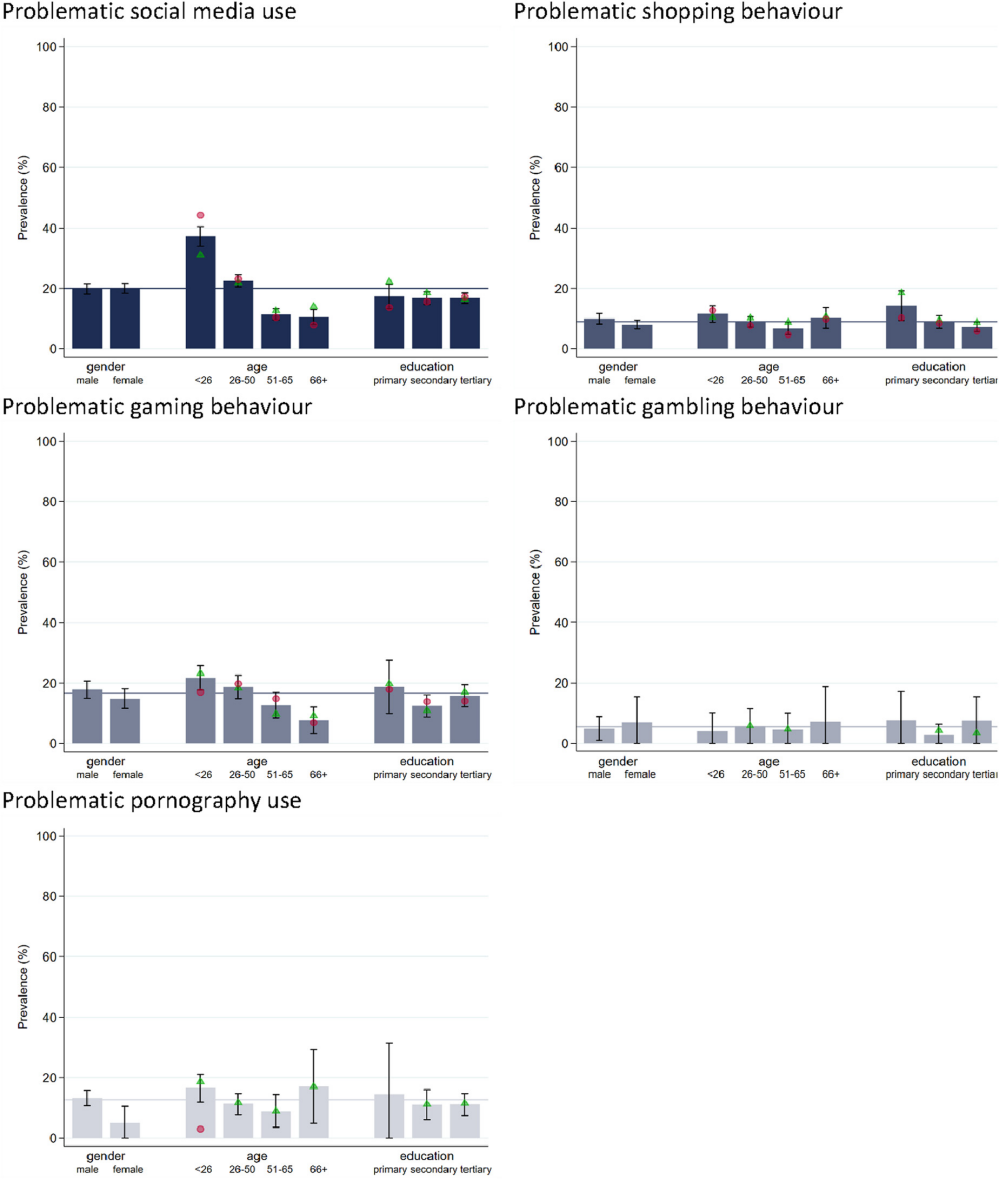
2023: Prevalences of the Problematic Behaviour Scale (PBS-5; at least weekly users within the last 30 days) by behaviour, and gender, age and education *Note:* Prevalences are nationally representative using sample weights. Whiskers indicate 95-% confidence intervals. Gender differences in age, and education: green triangle = men, red circle = women. Values with less than 30 cases are suppressed. For a more detailed description of the variables see Table A1 in the Appendix. For the repartition by education, age is restricted to >25 years, for which completed educational careers are assumed. The horizontal line refers to the overall mean depicted in Fig. 4.

Figure 5 provides an overview of the repartition of the prevalences of problematic behaviours by gender, age, and education. Regarding gender, PBS-5 prevalences are higher for males than for females in gaming, and pornography use, while no substantial differences could be found in social media use, shopping, and gambling. Systematic differences regarding age are observed for social media use (to a large extent), and gaming: younger individuals show higher rates of problematic behaviour than older individuals. Educational differences are found for shopping, where individuals with primary education show higher levels of problematic behaviour than persons with secondary or tertiary education.

Within age groups, systematic gender differences are found for social media use: While for subjects aged less than 26 years females showed a substantially higher rate of problematic social media use than males, the opposite is true for individuals aged 66 or older. Young men aged less than 26 years also showed higher rates of problematic gaming and pornography use than young women aged less than 26 years. Within educational subgroups, systematic gender differences were observed for social media use, and shopping: Men with primary education showed higher rates of problematic social media use, and shopping than women with primary education. For secondary and tertiary education, no substantial gender differences in problematic social media use, and shopping were found.

### Study 2

The results of the *exploratory factor analysis* of PBS-5 and PHQ-4 based on the data of Health and Lifestyle 2025 are similar to those based on its 2023 edition. This also applies to *internal consistency*. Furthermore, the results of multiple OLS regressions of PHQ-4 on PBS-5 (*external validation analysis*) are also the same (detailed results available upon request).

#### Cut-offs

ROC-regressions of the PHQ-4 dummy based on the 2025 data yield similar results as compared to those based on the 2023 data. This highlights the stability of the cut-off of the normalized values of PBS-5 of 20 for social media use, shopping, gaming, and pornography use (see Table S4 in the SI). However, for gambling the highest AUC is retained for a cut-off value of 20 in 2025 (AUC = .66), which deviates from the 2023 result with a suggested cut-off value of 50. Yet, the 2025 AUC of the cut-off value 50 (AUC = .64) is very close to the AUC of the cut-off value 20 and these AUCs are statistically not distinguishable. This ambiguous finding for gambling is probably the consequence of the low number of observations. For reasons of comparability over time, the national prevalence of problematic gambling reported below is based on the cut-off value of 50.

#### National prevalence rates of use frequency and problematic behaviour 2023 and 2025 in comparison

**Fig. 6.**
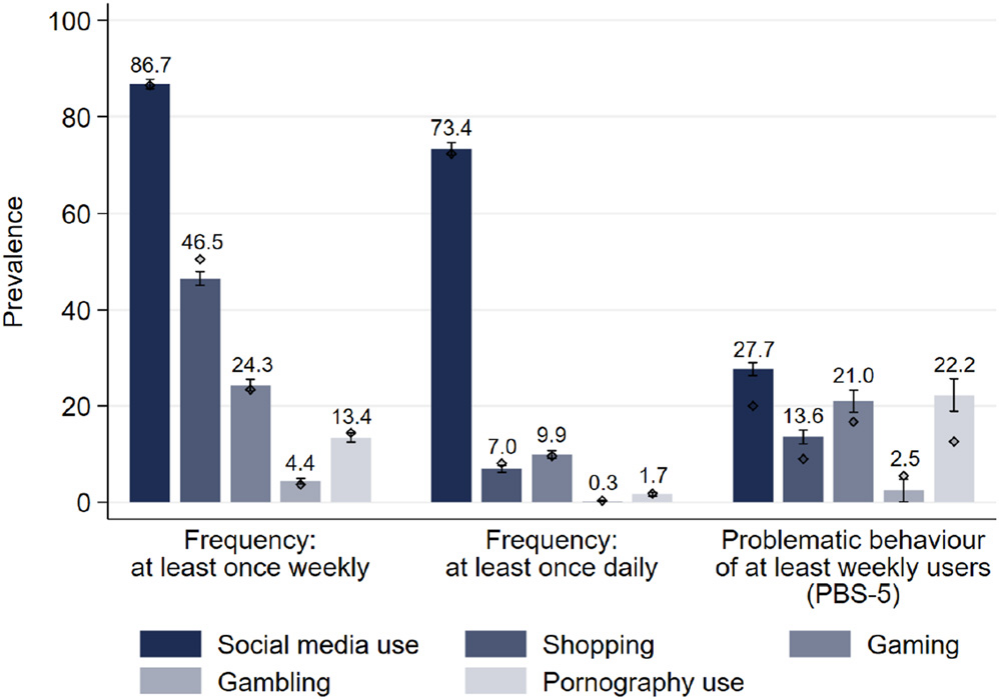
2025: Prevalences of the frequencies of behavioural conduct (within the last 30 days) and the Problematic Behaviour Scale (PBS-5; at least weekly users within the last 30 days) *Note:* Prevalences are nationally representative using sample weights. Whiskers indicate 95-% confidence intervals. Black diamond = 2023. For a more detailed description of the variables see Table S1 in the SI.

**Fig. 7.**
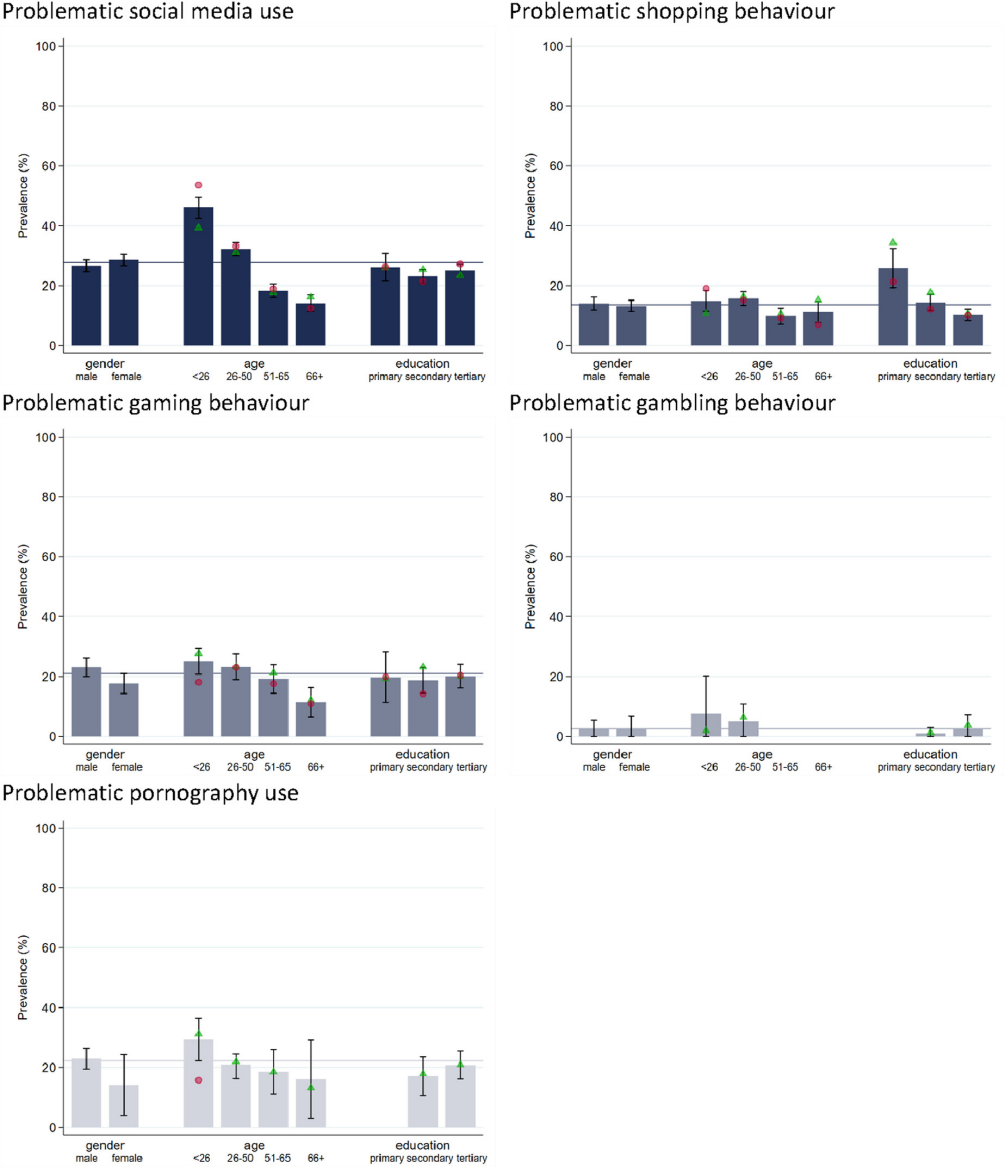
2025: Prevalences of the Problematic Behaviour Scale (PBS-5; at least weekly users within the last 30 days) by behaviour, and gender, age and education *Note:* Prevalences are nationally representative using sample weights. Whiskers indicate 95-% confidence intervals. Gender differences in age, and education: green triangle = men, red circle = women. Values with less than 30 cases are suppressed. For a more detailed description of the variables see Table S1 in the SI. For the repartition by education, age is restricted to >25 years, for which completed educational careers are assumed. The horizontal line refers to the overall mean depicted in Fig. 6.

Figure 6 shows that the prevalence rates of use frequency have been rather stable between 2023 and 2025 across behaviours except for shopping. The prevalence of shopping least once weekly decreased from 50.5% in 2023 to 45.5% in 2025. In 2025, higher prevalences as compared to 2023 are observed for problematic social media use (27.7%, +7.7%-points), problematic shopping (13.6%, +4.6%-points), problematic gaming (21.0%, +4.3%-points), and problematic pornography use (22.2%, +9.6%-points) (see Fig. 6). However, the statistical uncertainty of the point estimate for the prevalence of problematic pornography use in 2025 is relatively high. This applies even more to the prevalence rates of problematic gambling in 2023 and 2025 due to a relatively low number of observations. Hence, statements on a substantial trend for problematic gambling could not be inferred from the two samples of the survey Health and Lifestyle 2023 and 2025. Repartitions of the prevalences of problematic behaviours by gender, age, and education in 2025 (see Fig. 7) highlight the differences reported for 2023 (see Fig. 5), except for gender differences within educational subgroups. While in 2023, men with primary education showed higher rates of problematic social media use than women with primary education this difference is not observed in 2025.

## Discussion and conclusions

The PBS-5 is a promising brief screening tool for problematic behavioural conduct for social media use, shopping, gaming, gambling, and pornography use (online and offline) in German, French and Italian in the Swiss context. The longer PBS-10 does not outperform PBS-5 in any of the tested characteristics (for the results of similar analyses for PBS-10 please consult the online SI of the article). Hence, our findings are in line with previous studies on psychometric characteristics of ACSID-11 (Huang et al., 2025; Müller et al., 2022; Oelker et al., 2024; Yang et al., 2023).

The very high factor loadings, and excellent internal consistency of PBS-5 across the five analyzed behaviours suggest that the scale primarily captures general problem severity and thus functions as transdiagnostic severity index, which may not capture behaviour-specific symptomatologic heterogeneity. Moreover, very high factor loadings, and excellent internal consistency of PBS-5 suggest item redundancy. Hence, we also conducted our analyses with the general single items for functional impairment in life (FI1) or marked distress in life (MD1) or the 3-item scale consisting of the items representing the dimensions impaired control (IC3), increased priority (IP3), and continuation/escalation (CE3). The results reveal a substantially worse performance as compared to PBS-5 (results available upon request). Hence, with PBS-5 we were able to identify a promising short screening instrument of general problem severity for behavioural addictions for population-wide surveys.

Prevalence rates of problematic behaviours based on the here proposed cut-offs of PBS-5 only partly compare to other national data using other established screening instruments for behavioural addictions. However, these instruments are based on different theoretical dimensions and cut-off calculations (see Table 7 for an overview) and prevalence rates are thus not directly comparable. For shopping and gambling prevalence rate estimates are similar, while for social media use and gaming the prevalences differ to a great extent. The individual differences and similarities by gender, age, and education found with PBS-5 diverge partly from other instruments. Thus, the present study extends the available evidence on differences in problematic behaviours between sociodemographic and socioeconomic subgroups.

**Table 7.** Comparison of prevalences of problematic behaviour based on PBS-5 and alternative scales

Behaviour	PBS-5			Alternative screening instruments			
	Prevalence of problematic behaviour (in %)	Age range	Data source	Prevalence of problematic behaviour (in %)	Scale	Age range	Data source
Social media	35.5	15	SHL 2023	6.9	SMDS	15	HBSC 2022
Shopping	9.0	15+	SHL 2023	7.8	BSAS	15+	SHL 2022
Gaming	15.8	14–15	SHL 2023	2.8	IGDT	14–15	HBSC 2022
Gambling	5.5	15+	SHL 2023	4.3	Lie/Bet – NODS-CLiP	15+	SHS 2022
Pornography	12.6	15+	SHL 2023	n.a.	n.a.	n.a.	n.a.

*Note:* Prevalences are in % of the population. Prevalences are nationally representative using sample weights. BSAS = Bergen Shopping Addiction Scale (Andreassen et al., 2015). HBSC 2022 = Health Behaviour in School-aged Children 2022 (Addiction Switzerland, 2023). IGDT = Internet Gaming Disorder Test (Király et al., 2017). Lie/Bet – NODS-CLiP (Dey & Haug, 2019). SHL 2022 = Survey Health and Lifestyle 2022 (SFOPH, 2023). SHL 2023 = Survey Health and Lifestyle 2023 (SFOPH, 2024). SHS 2022 = Swiss Health Survey 2022 (SFSO, 2024). SMDS = Social Media Disorder Scale (Van den Eijnden, Lemmens, & Valkenburg, 2016). n.a. = not available.

The data and methods used in this study have several strengths: First, the Swiss survey “Health and Lifestyle” provides nationally representative repeated cross-sectional data of high quality in three languages (DE, FR, and IT) for a broad age range (15 years +), with high statistical power, and comparability across survey waves. Second, we use ROC regression to determine cut-off values of PBS-5 data-driven while controlling for potential confounders. The benefit of ROC regression is that it tests the quality of classification based on the confusion matrix simultaneously minimizing Type I (*α*, false positives) and Type II (*β*; false negatives) error of classification, i.e., maximizing specificity (true negatives) and sensitivity (true positives). Recall that conventional OLS regression only minimizes Type I error. Based on model fit (AUC) ROC regression also enables the comparison of classification quality of different cut-off values based on data. Furthermore, inclusion of PBS-5 and PHQ-4 in the Health and Lifestyle editions 2023 and 2025 allowed investigating the intertemporal stability of the suggested data-driven cut-off determination approach.

Hence, we follow a phenomenological approach determining cut-off values for problematic addictive behaviours based on empirical data. This approach contrasts theory-driven cut-off determination (e.g., Oelker et al., 2024) for instance following WHO guidelines (c.f. introduction). The cut-off procedure followed by Oelker et al. (2024) reduces variance from the beginning without empirically testing the validity of the theoretical categorization on the observed sample. In the absence of a clinical validation study a data-driven cut-off determination approach could be a reasonable alternative to a theory-driven approach. This is also underscored by the fact that the data-derived cut-offs outperform cut-offs as proposed by Oelker et al. (2024) with regard to ROC-based AUCs and their uncertainty, i.e., standard errors (irrespective of PBS-5 or PBS-10 as basis; see Table 8).

**Table 8.** Multiple receiver operator characteristic (ROC) regressions of PHQ-4 on PBS-5 and PBS-10

Models	(1)	(2)
Dependent variable	PHQ-4 (dummy with cut-off at >50)	
Independent variable	PBS-5 (dummy with cut-off as proposed by Oelker et al., 2024)	PBS-10 (dummy with cut-off as proposed by Oelker et al., 2024)
Social media	0.39 (0.02)	0.44 (0.02)
*n*	4,932	4,932
Shopping	0.54 (0.07)	0.49 (0.05)
*n*	2,815	2,815
Gaming	0.52 (0.13)	0.44 (0.07)
*n*	1,451	1,451
Gambling	0.59 (0.22)	0.61 (0.23)
*n*	175	175
Pornography	0.60 (0.30)	0.60 (0.06)
*n*	818	818

*Note:* Area under the curve (AUC) of multiple ROC-regression including standard errors in parentheses based on 100 bootstrap replications. The model includes the dummy of PBS-5 of each behaviour (social media, shopping, gaming, gambling, or pornography) separately. Each model controls for gender, age, education and survey language.

Therefore, we propose the application of an empirical ROC regression-based procedure to determine cut-offs in absence of clinically validated cut-offs using reliable and valid diagnostic instruments for depression and anxiety as well as other relevant external validation criteria as a potential alternative to theory-driven cut-off determination. Thus, we contribute to the ongoing discussion in addiction research on the relative value of objective, expert evaluations vs. subjective evaluations to determine service need (e.g., Hyshka et al., 2017).

Still, the PBS-5 as well as our study have some important limitations: The PBS-5 instrument was only administered to participants reporting at least weekly use within the last 30 days. This limits the generalizability of our findings to regular users and may confine the stability of estimates in small subsamples (e.g., gambling). Moreover, the data we use is cross-sectional. Hence, we cannot investigate the predictive validity of PBS-5 at time *t* regarding PHQ-4 at *t + 1* or difference-in-differences analyses based on longitudinal panel/cohort data. Future research may provide further insight regarding the predictive validity of the PBS-5. Generally, non-random non-response due to the voluntariness of survey participation and high sensitivity associated with addictive behaviours may bias estimates. Furthermore, the external validity of PBS-5 regarding self-reported symptoms of mental disorders (PHQ-4) is low to modest and the ability of PBS-5 to maximize sensitivity and specificity (ROC regression) regarding PHQ-4 is limited. Therefore, prevalence estimates based on our approach may be biased. Moreover, PHQ-4 exclusively captures internalizing symptomatology. Disorders due to addictive behaviours like gambling or gaming are often characterized by both internalizing and externalizing symptomatology. This may contribute to low to modest AUC values observed in the ROC analyses. Thus, relying exclusively on PHQ-4 for cut-off-determination may result in an underestimation of prevalence rates of problematic behaviour. Hence, future research may address clinical validation of the PBS-5 and its applicability for further behaviours utilizing more elaborate, gold standard instruments to capture external validation criteria like mental disorders, addictive behaviour or treatment seeking. Yet, PHQ-4 is a very efficient screening instrument for important mental disorders (anxiety and depression), and its reliability and external validity has been demonstrated in various settings and populations (e.g., Adzrago et al., 2024; Kroenke et al., 2009; Wicke et al., 2022). Additionally, while factor loadings are similar across survey languages, measurement invariance was not formally assessed. Future research may investigate the measurement invariance of PBS-5 across groups (e.g., genders, languages, countries).

In conclusion, PBS-5 is a promising brief screening instrument for behavioural addictions for population-wide surveys providing a common theoretical framework bridging various behaviours.

## Supplementary material

## Data availability

The data used in this study is available for scientific use upon request to the Swiss Federal Office of Public Health.

## Data Availability

The data used in this study is available for scientific use upon request to the Swiss Federal Office of Public Health.

## Appendix

**Table A1.** Health and Lifestyle 2023: Descriptive statistics of variables

Variable	Description	mean	sd	min.	max.	*n*
Frequency	Dummies, 1 for usage frequency of at least once a week in the past 30 days					
Social media use		0.865	0.005	0	1	5,989
Shopping		0.505	0.007	0	1	5,988
Gaming		0.234	0.006	0	1	5,987
Gambling		0.037	0.003	0	1	5,988
Pornography use		0.144	0.005	0	1	5,984
Frequency	Dummies, 1 for usage frequency of at least once daily in the past 30 days					
Social media use		0.724	0.007	0	1	5,989
Shopping		0.081	0.004	0	1	5,988
Gaming		0.096	0.004	0	1	5,987
Gambling		0.004	0.001	0	1	5,988
Pornography use		0.018	0.002	0	1	5,984
Problematic Behaviour Scale (PBS-5)	See Tables 2 and 3, as adapted from Müller et al. (2022)					
Social media use		7.262	0.045	5	25	5,295
Shopping		6.051	0.048	5	25	3,055
Gaming		6.876	0.091	5	25	1,585
Gambling		7.077	0.337	5	25	197
Pornography use		6.556	0.111	5	25	900
PBS-5 normalized	Normalized score of PBS-5 (0–100)					
Social media use		11.311	0.227	0	100	5,295
Shopping		5.255	0.241	0	100	3,055
Gaming		9.381	0.453	0	100	1,585
Gambling		10.384	1.684	0	100	197
Pornography use		7.779	0.553	0	100	900
PBS-5 dummies						
Social media use	Dummy, 1 for normalized score of PBS-5 > 20	0.200	0.006	0	1	5,295
Shopping	Dummy, 1 for normalized score of PBS-5 > 20	0.090	0.006	0	1	3,055
Gaming	Dummy, 1 for normalized score of PBS-5 > 20	0.167	0.011	0	1	1,585
Gambling	Dummy, 1 for normalized score of PBS-5 > 50	0.055	0.018	0	1	197
Pornography use	Dummy, 1 for normalized score of PBS-5 > 20	0.126	0.012	0	1	900
Patient Health Questionnaire-4 (PHQ-4)	PHQ-4 (Kroenke et al., 2009): Four-item sum scale for the screening of symptoms of depression and anxiety	5.770	0.036	4	16	5,980
PHQ-4 normalized	Normalized score of PHQ-4 (0–100)	14.747	0.299	0	100	5,980
PHQ-4 dummy	Dummy, 1 for normalized score of PHQ-4 > 50	0.067	0.003	0	1	5,980
Gender: female	Dummy, 1 for females	0.505	0.007	0	1	5,995
Age	In years	48.525	0.271	15	100	5,995
Education						
Low	Dummy, 1 for participants with primary education	0.155	0.005	0	1	5,988
Middle	Dummy, 1 for participants with secondary education	0.375	0.007	0	1	5,988
High	Dummy, 1 for participants with tertiary education	0.470	0.007	0	1	5,988
Survey language						
German	Dummy, 1 for German survey language	0.687	0.007	0	1	5,995
French	Dummy, 1 for French survey language	0.248	0.006	0	1	5,995
Italian	Dummy, 1 for Italian survey language	0.065	0.003	0	1	5,995

*Note:* Means are nationally representative using sample weights.

**Table A2.** Health and Lifestyle 2023: Correlation matrix of frequencies of behavioural conduct and PBS-5

Pearson's correlation coefficient		(1)	(2)	(3)	(4)	(5)	(6)	(7)	(8)	(9)	(10)
(1)	Frequency social media use	1 (5,989)									
(2)	Frequency shopping	.28*** (5,988)	1 (5,988)								
(3)	Frequency gaming	.15*** (5,987)	.10*** (5,987)	1 (5,987)							
(4)	Frequency gambling	.01 (5,988)	.09*** (5,987)	.12*** (5,987)	1 (5,988)						
(5)	Frequency porn use	.13*** (5,984)	.08*** (5,984)	.24*** (5,984)	.14*** (5,984)	1 (5,984)					
(6)	PBS-5_social media_	.16*** (5,295)	.13*** (5,295)	.10*** (5,295)	.02 (5,295)	.13*** (5,294)	1 (5,295)				
(7)	PBS-5_shopping_	−.01 (3,055)	.17*** (3,055)	.02 (3,055)	.09*** (3,055)	−.00 (3,052)	.51*** (2,885)	1 (3,055)			
(8)	PBS-5_gaming_	−.01 (1,585)	.09*** (1,585)	.17*** (1,585)	.10*** (1,585)	.16*** (1,585)	.56*** (1,490)	.45*** (884)	1 (1,585)		
(9)	PBS-5_gambling_	−.09 (197)	.17* (197)	.08 (197)	.37*** (197)	.03 (197)	.58*** (179)	.68*** (127)	.60*** (86)	1 (197)	
(10)	PBS-5_porn_	.02 (900)	.06 (900)	.08* (900)	.06 (900)	.21*** (900)	.43*** (851)	.37*** (530)	.44*** (434)	.79*** (63)	1 (900)

*Note:* Pearson's correlation coefficients handling missing values by pairwise deletion. * = *p* < 0.05, ** = *p* < 0.01, *** = *p* < 0.001. Numbers in parentheses indicate the number of observations.

**Table A3.** Health and Lifestyle 2023: The Problematic Behaviour Scale (PBS-5): Social media use

				all	DE	FR	IT
I	(1)		How often have you tried to stop or restrict the activity and failed with it?	.81	.82	.79	.81
	(2)		How often have you neglected or given up other activities or interests that you used to enjoy because of the activity?	.83	.84	.82	.83
	(3)		How often have you continued or increased the activity even though it caused you physical or mental complaints/diseases?	.85	.86	.82	.87
	(4)		If you think about all areas of your life, how often has your life been noticeably affected by the activity?	.88	.90	.86	.86
	(5)		If you think about all areas of your life, how often did the activity cause you suffering?	.87	.88	.87	.87
*n*				5,295	3,429	1,289	577
Percent of variance explained by factor I				72.3	73.9	69.2	71.8
Cronbach's *α*				.83	.84	.78	.82
PBS-5:		mean		7.53	7.64	7.13	7.80
		SD		3.19	3.22	2.98	3.38
		min		5	5	5	5
		max		25	24	25	25

*Note:* Numbers indicate factor loadings after orthogonal varimax-rotated exploratory factor analysis (principal component analysis (PCA)) based on polychoric correlations for ordinal data. The criterion for factor extraction is an eigenvalue greater than 1. An equivalent analysis with simple Pearson's correlations yields substantially similar results. DE = German, FR = French, IT = Italian.

**Table A4.** Health and Lifestyle 2023: The Problematic Behaviour Scale (PBS-5): Shopping

			all	DE	FR	IT
I	(1)	How often have you tried to stop or restrict the activity and failed with it?	.87	.88	.85	.87
	(2)	How often have you neglected or given up other activities or interests that you used to enjoy because of the activity?	.92	.92	.92	.95
	(3)	How often have you continued or increased the activity even though it caused you physical or mental complaints/diseases?	.93	.95	.89	.93
	(4)	If you think about all areas of your life, how often has your life been noticeably affected by the activity?	.93	.95	.89	.92
	(5)	If you think about all areas of your life, how often did the activity cause you suffering?	.94	.95	.91	.92
*n*			3,055	1,968	784	303
Percent of variance explained by factor I			84.6	86.4	79.8	84.3
Cronbach's *α*			.88	.90	.81	.88
PBS-5:		mean	6.10	6.21	5.76	6.30
		SD	2.40	2.50	1.96	2.67
		min	5	5	5	5
		max	25	25	25	20

*Note:* Numbers indicate factor loadings after orthogonal varimax-rotated exploratory factor analysis (principal component analysis (PCA)) based on polychoric correlations for ordinal data. The criterion for factor extraction is an eigenvalue greater than 1. An equivalent analysis with simple Pearson's correlations yields substantially similar results. DE = German, FR = French, IT = Italian.

**Table A5:** Health and Lifestyle 2023: The Problematic Behaviour Scale (PBS-5): Gaming

			all	DE	FR	IT
I	(1)	How often have you tried to stop or restrict the activity and failed with it?	.85	.86	.81	.87
	(2)	How often have you neglected or given up other activities or interests that you used to enjoy because of the activity?	.89	.89	.91	.91
	(3)	How often have you continued or increased the activity even though it caused you physical or mental complaints/diseases?	.89	.90	.86	.95
	(4)	If you think about all areas of your life, how often has your life been noticeably affected by the activity?	.91	.94	.87	.95
	(5)	If you think about all areas of your life, how often did the activity cause you suffering?	.91	.92	.89	.92
*n*			1,585	1,036	402	147
Percent of variance explained by factor I			79.2	81.6	75.4	84.5
Cronbach's *α*			.87	.88	.81	.90
PBS-5:		mean	7.00	6.92	6.97	7.61
		SD	3.12	3.00	3.22	3.58
		min	5	5	5	5
		max	25	23	25	20

*Note:* Numbers indicate factor loadings after orthogonal varimax-rotated exploratory factor analysis (principal component analysis (PCA)) based on polychoric correlations for ordinal data. The criterion for factor extraction is an eigenvalue greater than 1. An equivalent analysis with simple Pearson's correlations yields substantially similar results. DE = German, FR = French, IT = Italian.

**Table A6.** Health and Lifestyle 2023: The Problematic Behaviour Scale (PBS-5): Gambling

			all	DE	FR	IT
I	(1)	How often have you tried to stop or restrict the activity and failed with it?	.88	.94	.	.
	(2)	How often have you neglected or given up other activities or interests that you used to enjoy because of the activity?	.92	.96	.	.
	(3)	How often have you continued or increased the activity even though it caused you physical or mental complaints/diseases?	.93	.97	.	.
	(4)	If you think about all areas of your life, how often has your life been noticeably affected by the activity?	.96	.98	.	.
	(5)	If you think about all areas of your life, how often did the activity cause you suffering?	.92	.96	.	.
*n*			197	121	60	16
Percent of variance explained by factor I			93.0	92.5	.	.
Cronbach's *α*			.95	.95	.	.
PBS-5:		mean	7.08	6.97	.	.
		SD	4.01	3.70	.	.
		min	5	5	.	.
		max	25	20	.	.

*Note:* Numbers indicate factor loadings after orthogonal varimax-rotated exploratory factor analysis (principal component analysis (PCA)) based on polychoric correlations for ordinal data. The criterion for factor extraction is an eigenvalue greater than 1. An equivalent analysis with simple Pearson's correlations yields substantially similar results. DE = German, FR = French, IT = Italian.

**Table A7.** Health and Lifestyle 2023: The Problematic Behaviour Scale (PBS-5): Pornography use

			all	DE	FR	IT
I	(1)	How often have you tried to stop or restrict the activity and failed with it?	.85	.87	.82	.
	(2)	How often have you neglected or given up other activities or interests that you used to enjoy because of the activity?	.88	.88	.85	.
	(3)	How often have you continued or increased the activity even though it caused you physical or mental complaints/diseases?	.90	.90	.89	.
	(4)	If you think about all areas of your life, how often has your life been noticeably affected by the activity?	.92	.93	.90	.
	(5)	If you think about all areas of your life, how often did the activity cause you suffering?	.93	.92	.96	.
*n*			900	587	225	88
Percent of variance explained by factor I			80.3	81.4	78.2	.
Cronbach's *α*			.84	.85	.82	.
PBS-5:		mean	6.67	6.67	6.79	.
		SD	2.91	2.88	3.07	.
		min	5	5	5	.
		max	25	25	25	.

*Note:* Numbers indicate factor loadings after orthogonal varimax-rotated exploratory factor analysis (principal component analysis (PCA)) based on polychoric correlations for ordinal data. The criterion for factor extraction is an eigenvalue greater than 1. An equivalent analysis with simple Pearson's correlations yields substantially similar results. DE = German, FR = French, IT = Italian.

**Table A8.** Health and Lifestyle 2023: Multiple OLS regressions of PHQ-4 on PBS-5

Model	(1)	(2)	(3)	(4)	(5)
Dependent variable	PHQ-4 (z-standardized)				
Behaviour	Social media use	Shopping	Gaming	Gambling	Pornography use
PBS-5 (z-stand. index)	0.40***	0.26***	0.32***	0.43***	0.32***
	(0.02)	(0.02)	(0.03)	(0.10)	(0.04)
Gender: female	0.18***	0.23***	0.46***	0.14	0.91***
	(0.02)	(0.04)	(0.05)	(0.19)	(0.13)
Age	−0.01***	−0.01***	−0.01***	−0.01	−0.01***
	(0.00)	(0.00)	(0.00)	(0.01)	(0.00)
Education: middle	0.03	0.00	0.08	0.07	0.01
	(0.04)	(0.06)	(0.07)	(0.19)	(0.10)
Education: high	0.05	0.08	0.19*	0.37	0.05
	(0.04)	(0.06)	(0.08)	(0.22)	(0.10)
Survey language: French	0.15***	0.17***	0.10	0.33	0.09
	(0.03)	(0.04)	(0.06)	(0.17)	(0.08)
Survey language: Italian	−0.08*	−0.10	−0.11	0.29	−0.21
	(0.04)	(0.06)	(0.07)	(0.27)	(0.11)
Constant	0.16***	0.46***	0.35***	0.23	0.45***
	(0.04)	(0.06)	(0.06)	(0.20)	(0.10)
*n*	5,292	3,053	1,585	197	900
adj. *R*^2^	0.21	0.14	0.16	0.17	0.17

*Note:* Coefficients of multiple OLS-regressions including standard errors in parentheses based on 100 bootstrap replications. * = *p* < 0.05, ** = *p* < 0.01, *** = *p* < 0.001.
