# Supplementary material for: The Problematic Behaviour Scale (PBS-5): A brief measure for the population-level screening of non-substance-bound addictive behaviours and Swiss national prevalence rates
Source: J Behav Addict. 2026 Mar 20;15(1):209–29. doi: 10.1556/2006.2025.00120 (PMC13132348; doi:10.1556/2006.2025.00120)
Supplement: Supplementary file 1 [file jba-15-209-s001.pdf]

Sebastian Mader, S., & Simon Marmet, S.: The Problematic Behaviour Scale (PBS-5): A brief measure for the population-level screening of non-substance-bound addictive behaviours and Swiss national prevalence rates

https://doi.org/10.1556/2006.2025.00120

Supplementary materials

Table S1: Health and Lifestyle 2025: Descriptive statistics of variables

Variable	Description	mean	sd	min.	max.	n
Frequency	Dummies, 1 for usage frequency of at least once a week in the past 30 days					
Social media use		0.867	0.005	0	1	5808
Shopping		0.465	0.007	0	1	5809
Gaming		0.243	0.006	0	1	5807
Gambling		0.044	0.003	0	1	5806
Pornography use		0.134	0.005	0	1	5803
Frequency	Dummies, 1 for usage frequency of at least once daily in the past 30 days					
Social media use		0.734	0.006	0	1	5808
Shopping		0.070	0.004	0	1	5809
Gaming		0.099	0.004	0	1	5807
Gambling		0.003	0.001	0	1	5806
Pornography use		0.017	0.002	0	1	5803
Problematic Behaviour Scale (PBS-5)	See Tables 2 and 3, as adapted from Müller et al. (2022)					
Social media use		8.024	0.052	5	25	5127
Shopping		6.565	0.057	5	25	2724
Gaming		7.364	0.091	5	25	1605
Gambling		6.941	0.241	5	25	243
Pornography use		7.707	0.155	5	25	759
PBS-5 normalized	Normalized score of PBS-5 (0-100)					
Social media use		15.121	0.260	0	100	5127
Shopping		7.824	0.284	0	100	2724
Gaming		11.819	0.456	0	100	1605
Gambling		9.705	1.205	0	100	243
Pornography use		13.537	0.776	0	100	759
PBS-5 dummies						
Social media use	Dummy, 1 for normalized score of PBS-5 > 20	0.277	0.007	0	1	5127
Shopping	Dummy, 1 for normalized score of PBS-5 > 20	0.136	0.007	0	1	2724
Gaming	Dummy, 1 for normalized score of PBS-5 > 20	0.210	0.012	0	1	1605
Gambling	Dummy, 1 for normalized score of PBS-5 > 50	0.025	0.012	0	1	243
Pornography use	Dummy, 1 for normalized score of PBS-5 > 20	0.222	0.017	0	1	759
Patient Health Questionnaire-4 (PHQ-4)	PHQ-4 (Kroenke et al. 2009): Four-item sum scale for the screening of symptoms of depression and anxiety.	5.652	0.032	4	16	5782
PHQ-4 normalized	Normalized score of PHQ-4 (0-100)	13.767	0.268	0	100	5782
PHQ-4 dummy	Dummy, 1 for normalized score of PHQ-4 > 50	0.045	0.003	0	1	5782
gender: female	Dummy, 1 for females	0.504	0.007	0	1	5818
Age	In years	48.623	0.275	15	97	5818
Education						
Low	Dummy, 1 for participants with primary education	0.169	0.005	0	1	5808
Middle	Dummy, 1 for participants with secondary education	0.382	0.007	0	1	5808
High	Dummy, 1 for participants with tertiary education	0.449	0.007	0	1	5808
Survey language						
German	Dummy, 1 for German survey language	0.687	0.007	0	1	5818
French	Dummy, 1 for French survey language	0.248	0.006	0	1	5818
Italian	Dummy, 1 for Italian survey language	0.066	0.003	0	1	5818

Note: means are nationally representative using sample weights.

Results of confirmatory factor analyses for a one-factorial solution of PBS-5 by behaviour

Figure S1: Structural Equation Model (SEM) of Confirmatory Factor Analyses (CFA) for the Problematic Behaviour Scale (PBS-5) and the Patient Health Questionnaire (PHQ-4)

PBS-5:

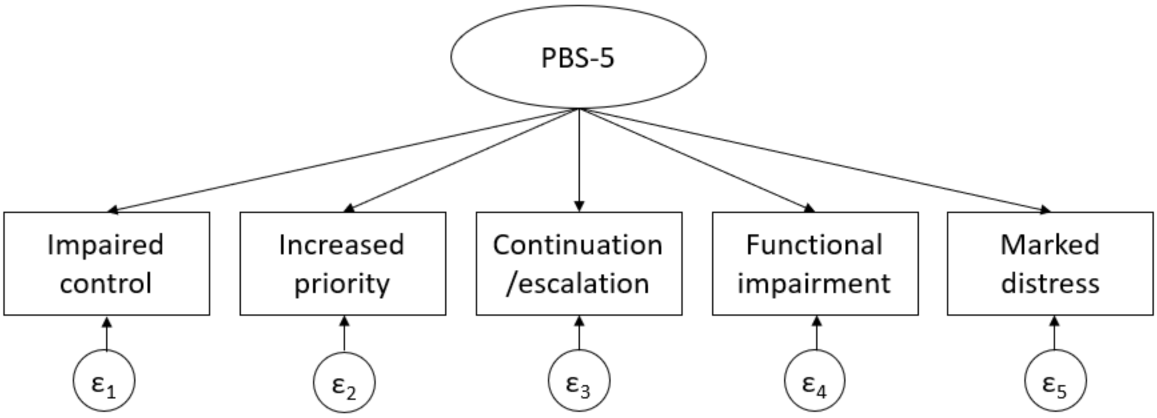

Note: For the detailed results see Table S2.

PHQ-4:

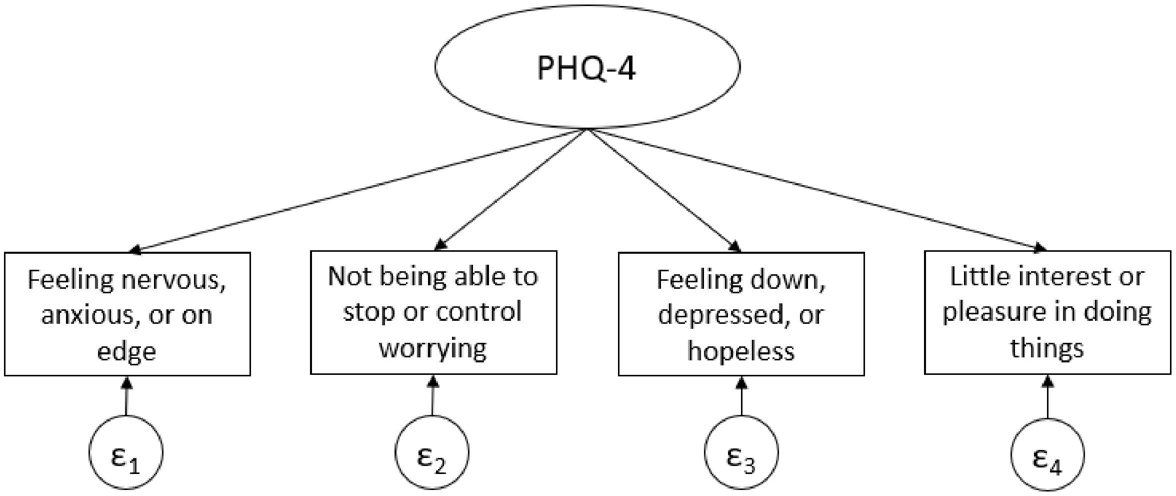

Note: For the detailed results see Table S3.

Table S2: SEM of CFA for PBS-5

Item	Trait Loading				
	Social media	Shopping	Gaming	Gambling	Porn
(1) How often have you tried to stop or restrict the activity and failed with it?	.69	.71	.65	.81	.66
(2) How often have you neglected or given up other activities or interests that you used to enjoy because of the activity?	.71	.80	.76	.88	.67
(3) How often have you continued or increased the activity even though it caused you physical or mental complaints/ diseases?	.75	.83	.89	.87	.72
(4) If you think about all areas of your life, how often has your life been noticeably affected by the activity?	.70	.79	.85	.98	.86
(5) If you think about all areas of your life, how often did the activity cause you suffering?	.69	.80	.74	.91	.83
n	5299	3056	1585	197	901
Model Fit:					
Likelihood Ratio Test (model vs. saturated)	$\chi^2(1)$=30.3, *p*=.000	$\chi^2(1)$=0.49, *p*=.486	$\chi^2(1)$=8.33, *p*=.004	$\chi^2(1)$=6.93, *p*=.009	$\chi^2(1)$=0.78, *p*=.377
Root Mean Squared Error of Approximation (RMSEA) [90% CI]	.074 [.05, .10]	.000 [.00, .04]	.068 [.03, .11]	.174 [.07, .31]	.000 [.00, .08]
Comparative Fit Index (CFI)	.997	1.000	.998	.995	1.000

Note: The analysis was conducted using the command 'sem' of the statistical software STATA 19.0. Factor loadings are standardized regression loadings. Measurement errors of items 1, 2, and 3 (representing the three main theoretical dimensions of disorders due to addictive behaviours) as well as of items 4 (marked distress as a consequence of the three main theoretical dimensions) and 5 (functional impairment in life as a consequence of the three main theoretical dimensions) are allowed to covary.

Table S3: SEM of CFA for PHQ-4

	Item	Trait Loading
(1)	Feeling nervous, anxious, or on edge	.73
(2)	Not being able to stop or control worrying	.69
(3)	Feeling down, depressed, or hopeless	.91
(4)	Little interest or pleasure in doing things	.78
	n	5986
	Model Fit:	
	Likelihood Ratio Test (model vs. saturated)	$\chi^2(1)$=7.13, *p*=.008
	Root Mean Squared Error of Approximation (RMSEA) [90% CI]	.032 [.01, .06]
	Comparative Fit Index (CFI)	.999

Note: The analysis was conducted using the command 'sem' of the statistical software STATA 19.0. Factor loadings are standardized regression loadings. Measurement errors of items 1, and 2 are allowed to covary.

Health and Lifestyle 2025

Table S4: Health and Lifestyle 2025: Multiple receiver operator characteristic (ROC) regressions of PHQ-4 on PBS-5

Model	(1)	(2)	(3)	(4)	(5)
Dependent variable	PHQ-4 (dummy with cut-off at >50)				
Independent variable	PBS-5 (dummy with cut-off at >20)	PBS-5 (dummy with cut-off at >40)	PBS-5 (dummy with cut-off at >50)	PBS-5 (dummy with cut-off at >60)	PBS-5 (dummy with cut-off at >80)
Social media	**0.61**	0.54	0.47	0.39	0.33
	**(0.02)**	(0.02)	(0.02)	(0.02)	(0.02)
n	4834	4834	4834	4834	4832
Shopping	**0.57**	0.44	0.42	0.40	0.42
	**(0.04)**	(0.04)	(0.04)	(0.04)	(0.17)
n	2551	2551	2551	2551	2551
Gaming	**0.62**	0.51	0.47	0.46	.
	**(0.04)**	(0.04)	(0.04)	(0.05)	.
n	1497	1497	1497	1497	.
Gambling	**0.66**	0.60	0.64	0.62	.
	**(0.08)**	(0.09)	(0.24)	(0.23)	.
n	221	221	221	221	.
Pornography	**0.66**	0.59	0.57	0.55	0.55
	**(0.05)**	(0.05)	(0.05)	(0.05)	(0.05)
n	701	701	701	701	701

Note: Area under the curve (AUC) of multiple ROC-regressions including standard errors in parentheses based on 100 bootstrap replications. Each model includes the dummy of the normalized values of PBS-5 of each behaviour (social media, shopping, gaming, gambling, or pornography) separately. Each model controls for gender, age, education and survey language. AUCs in bold font indicate the selected cut-offs.

Results of the analyses with the long 10-item Problematic Behaviour Scale (PBS-10)

Exploratory factor analysis and internal consistency:

The PCA generally retains a one-factorial solution with high factor loadings. The share of the variance explained by the factors ranges from 75 % for gaming to 90 % for gambling. The only exception is social media use, where the two factors impaired control/increased priority and escalation/ continuation are retained. Internal consistency of the PBS-10 is very high, and varies from α=.90 for social media to α=.97 for gambling (see Table S5).

Table S5: The Problematic Behaviour Scale (PBS-10): Exploratory factor analysis and internal consistency

	Item		Social media	Shopping	Gaming	Gambling	Porn
(1)	How often have you had problems controlling the activity (e.g., start, duration, intensity, situation, end)?		.86	.83	.83	.88	.84
(2)	How often have you felt the desire to stop or restrict the activity because you noticed you were using it too much?		.91	.85	.82	.92	.83
(3)	How often have you tried to stop or restrict the activity and failed with it?		.83	.90	.87	.94	.86
(4)	How often have you given the activity an increasingly higher priority than other activities or interests in your daily life?		.72	.91	.88	.97	.89
(5)	How often have you neglected or given up other activities or interests that you used to enjoy because of the activity?		.57	.92	.90	.96	.89
(6)	How often have you continued or increased the activity even though it has threatened or caused you to lose a relationship with someone important to you?		.85	.92	.88	.96	.86
(7)	How often have you continued or increased the activity even though it caused you problems at school / in training / at work?		.78	.92	.85	.94	.86
(8)	How often have you continued or increased the activity even though it caused you physical or mental complaints/diseases?		.85	.92	.86	.96	.87
(9)	If you think about all areas of your life, how often has your life been noticeably affected by the activity?		.70	.90	.89	.98	.89
(10)	If you think about all areas of your life, how often did the activity cause you suffering?		.73	.91	.86	.96	.90
n			5295	3055	1585	197	900
Percent of variance explained by empirical factors			77.6	80.8	74.7	89.9	75.5
Cronbach's α			.90	.93	.92	.97	.91
PBS-10:		Mean	16.02	12.60	14.82	14.43	13.98
		SD	6.46	4.86	6.33	7.94	5.87
		Min	10	10	10	10	10
		Max	50	50	50	50	50

Note: Introduction: "Please read the following questions carefully in relation to the activities you use. From the five possible answers, select the one that best describes how often you have had each experience in the last 30 days. There are no right or wrong answers. Now think about the activity "Social media use" / "Shopping" / "Gaming" / "Gambling" / "Use of pornography"." Answer categories: 1="never", 2="rarely", 3="sometimes", 4="often", 5="always". Numbers indicate factor loadings after orthogonal varimax-rotated exploratory factor analysis (principal component analysis (PCA)) based on polychoric correlations for ordinal data. The criterion for factor extraction is an eigenvalue greater than 1. An equivalent analysis with simple Pearson's correlations yields substantially similar results.

External Validity:

The external validation analysis shows that for each 1sd of PBS-10 PHQ-4 is between .27sd for shopping and .42sd for gambling higher (p<.001) (see Table S6).

Table S6: Multiple OLS regressions of PHQ-4 on PBS-10

Dependent variable	PHQ-4 (z-standardized)
Independent variable type	PBS-10 (z-stand. index)
Social media	0.40*** (0.02)
n	5292
adj. R^2	0.21
Shopping	0.27*** (0.02)
n	3053
adj. R^2	0.14
Gaming	0.33*** (0.03)
n	1585
adj. R^2	0.17
Gambling	0.42*** (0.10)
n	197
adj. R^2	0.17
Pornography	0.33*** (0.04)
n	900
adj. R^2	0.18

Note: Coefficients of multiple OLS-regressions including standard errors in parentheses based on 100 bootstrap replications. Each model includes the PBS-10 of each behaviour (social media, shopping, gaming, gambling, or pornography) separately. Each model controls for sex, age, education and survey language. * = p<0.05, ** = p<0.01, *** = p<0.001.

Cut-offs:

ROC-regression shows that the highest areas under the curve (AUC) for the tested cut-offs of the normalized PBS-10 scores (0-100) vary between AUC=.56 for shopping to AUC=.67 for pornography use (Table S7). Hence, a cut-off of the normalized values of PBS-10 of 20 is proposed for social media use, shopping, and gaming. A cut-off value of 50 is proposed for gambling and a cut-off of 40 is proposed for pornography use (while a cut-off of 20 has a comparable AUC). Figure S2 depicts the AUCs of the multiple ROC-regressions of these cut-offs.

Table S7: Multiple receiver operator characteristic (ROC) regressions of PHQ-4 on PBS-10

Models	(1)	(2)	(3)	(4)	(5)
Dependent variable	PHQ-4 (dummy with cut-off at >50)				
Independent variable type	PBS-10 (dummy with cut-off at >20)	PBS-10 (dummy with cut-off at >40)	PBS-10 (dummy with cut-off at >50)	PBS-10 (dummy with cut-off at >60)	PBS-10 (dummy with cut-off at >80)
Social media	**0.65**	0.58	0.50	0.45	0.48
	**(0.01)**	(0.02)	(0.02)	(0.02)	(0.19)
N	**4932**	4932	4932	4932	4932
Shopping	**0.56**	0.48	0.48	0.43	0.40
	**(0.03)**	(0.03)	(0.05)	(0.06)	(0.19)
N	**2815**	2815	2815	2815	2815
Gaming	**0.63**	0.57	0.51	0.55	.
	**(0.03)**	(0.04)	(0.05)	(0.05)	.
N	**1451**	1451	1451	1451	.
Gambling	0.61	0.62	**0.64**	0.54	0.63
	(0.10)	(0.10)	**(0.09)**	(0.16)	(0.32)
N	175	175	**175**	175	175
Pornography	0.65	**0.67**	0.61	0.59	.
	(0.04)	**(0.05)**	(0.06)	(0.13)	.
N	818	**818**	818	818	.

Note: Area under the curve (AUC) of multiple ROC-regressions including standard errors in parentheses based on 100 bootstrap replications. Each model includes the PBS-10 of each activity (social media, shopping, gaming, gambling, or pornography) separately. Each model controls for sex, age, education and survey language. AUC = area under the curve.

Problematic social media use (PBS-10 dummy with cut-off at normalized score of > 20)

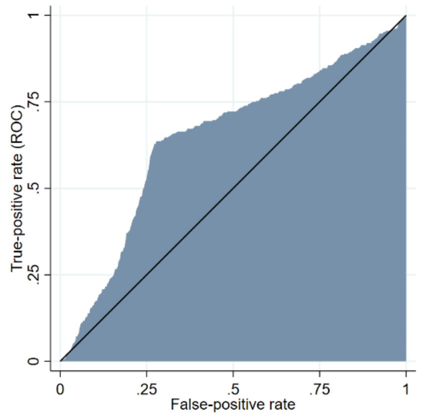

Problematic shopping behaviour (PBS-10 dummy with cut-off at normalized score of > 20)

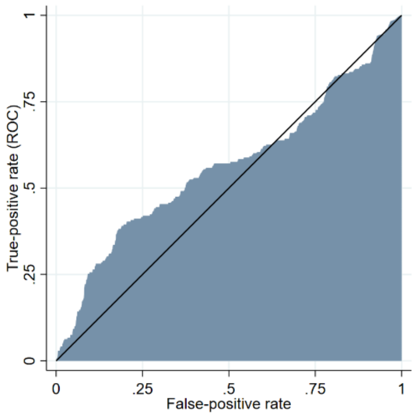

Problematic gaming behaviour (PBS-10 dummy with cut-off at normalized score of > 20)

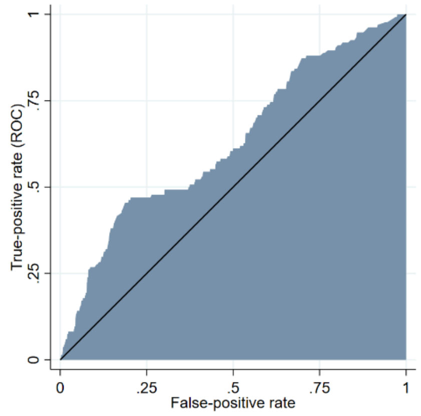

Problematic gambling behaviour (PBS-10 dummy with cut-off at normalized score of > 50)

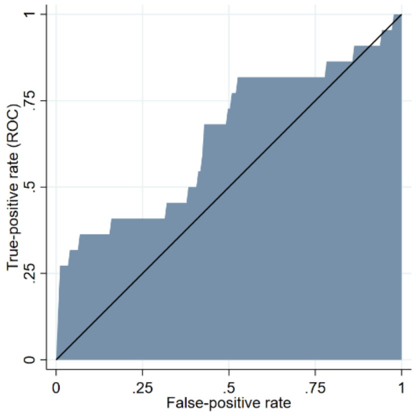

Problematic porn use (PBS-10 dummy with cut-off at normalized score of > 40)

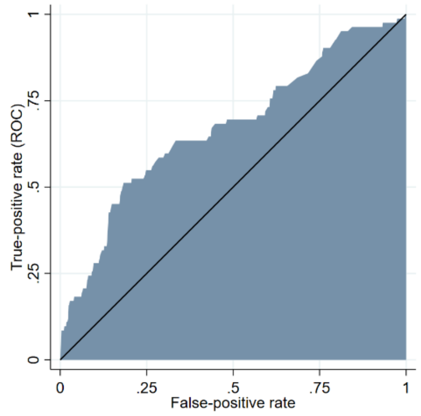

Figure S2: Area under the curve (AUC) of multiple ROC-regressions of PHQ-4 on PBS-10 (see Table S6)

Table S8 below describes the distributions of PBS-10.

Table S8: Description of variables

Variable	Description	mean	sd	min.	max.	n
Problematic Behaviour Scale (PBS-10)	See Tables 2 and 3, as adapted from Müller et al. (2022)					
Social media use		15.367	0.091	10	50	5295
Shopping		12.466	0.096	10	50	3055
Gaming		14.492	0.184	10	50	1585
Gambling		14.452	0.667	10	50	197
Pornography use		13.83	0.226	10	50	900
PBS-10 normalized	Normalized score of PBS-10 (0-100)					
Social media use		13.417	0.230	0	100	5295
Shopping		6.164	0.239	0	100	3055
Gaming		11.229	0.459	0	100	1585
Gambling		11.130	1.667	0	100	197
Pornography use		9.570	0.566	0	100	900

Note: means are nationally representative using sample weights.
